# Low birthweight in term singletons mediates the association between maternal smoking intensity exposure status and immediate neonatal intensive care unit admission: the E-value assessment

**DOI:** 10.1186/s12884-020-02981-1

**Published:** 2020-06-03

**Authors:** Anthony J. Kondracki

**Affiliations:** Department of Family Science, School of Public Health, Maternal and Child Health, University of Maryland, 4200 Valley Drive, College Park, MD 20742 USA

**Keywords:** Low birthweight, NICU admission, Term neonate, Smoking, Mediation, E-value

## Abstract

**Background:**

Research investigating the wellbeing of term neonates in the United States is scarce. The objectives of this study were to estimate the prevalence of low birthweight (LBW) and neonatal intensive care unit (NICU) admission among term singletons in association with maternal smoking intensity exposure status, to explore LBW as a mediator linking smoking to immediate newborn NICU transfer/admission, and to assess the potential impact of unmeasured confounding in effect estimates.

**Methods:**

The Natality File of live births registered in the United States in 2016, the first year that all 50 states implemented the revised 2003 standard birth certificate, was restricted to singleton term births (37–41 completed weeks gestation). The prevalence of LBW (< 2500 g) and NICU transfer/admission was estimated across maternal demographic characteristics and smoking intensity status in early and in late pregnancy. Mediation analyses, based on the counterfactual approach, were conducted to examine the total effect (TE), controlled direct effect (CDE), natural direct (NDE) and indirect effects (NIE), and the percentage mediated through LBW. The E-values based on effect size estimates and on lower-bounds of 95% confidence intervals (CIs) assessed the potential impact of unmeasured confounding.

**Results:**

Nearly 6.8% of women smoked in early and in late pregnancy, most (36.4%) smoked at high intensity (≥ 10 cigarettes /day) and had the highest prevalence of LBW (6.7%) and NICU transfer/admission (7.0%). For the largest smoking intensity exposure category, the estimate of the TE was 1.68 (95% CI: 1.63, 1.73), of the NDE was 1.56 (95% CI: 1.51, 1.61), of the NIE was 1.08 (95% CI:1.07, 1.09), and the percentage mediated by LBW was 17.6%. The E-values for association estimates and for the lower-bounds of 95% CIs demonstrated the minimum strength of the potential unmeasured confounding necessary to explain away observed associations.

**Conclusions:**

These findings fill a gap on the prevalence of LBW and NICU transfer/admission in term neonates of mothers who smoke and on the role of LBW linking to NICU placement, which could be used to update practitioners, to implement smoking cessation interventions, monitor trends, and to inform planning and allocation of healthcare resources.

## Background

In the United States, newborns are increasingly likely to be admitted to a neonatal intensive care unit (NICU). Data from time-trend analyses of nearly 18 million live births to US residents conducted in 38 states and the District of Columbia showed that between 2007 and 2012, the overall admission rate to the NICU increased from 64.0 to 77.9 per 1000 live births [1]. The need for NICU admission focused on the smallest and sickest infants delivered preterm (< 37 weeks gestation), nevertheless, during that period of time the number of term singletons in the NICU, who suffered unexpected newborn complications, [2] increased by 23% [1]. Birth weight depends on duration of gestation and intrauterine growth rate. Low birthweight (LBW) defined as less than 2500 g (or 5 pounds 8 oz) occurs in 30–60% of infants delivered preterm and has been associated with increased risk of mortality, short- and long-term morbidity, and economic burden [3–7]. Globally, an estimated 20 million infants are born every year with LBW [8]. In the United States, after a 4.0% increase since 2014, between 2017 and 2018, the overall rate of LBW remained stable at 8.3% and among singletons at about 6.6% [9].

Infants delivered at term (37 0/7 through 41 6/7 weeks gestation) [10] consist the largest birth cohort and are generally considered fully grown and healthy. Nevertheless, a proportion of term neonates may be born with a birth weight below the average normal weight between 2500 g (or 5 pounds 8 oz) and 4000 g (or 8 pounds 13 oz) and with morbidities that threaten their survival. Gestational age alone cannot explain LBW in term neonates, if not accounting for risk factors, such as smoking. Smoking is a common and preventable risk factor for preterm birth and LBW [11–15]. Small for gestational age, rather than preterm birth, has been suggested as the main mechanism through which smoking impacts infant mortality [16]. Despite recent declines, [17] the estimated high intensity smoking rate in the US in 2016 was 9.4% before pregnancy and 7.1% during pregnancy [18]. Tobacco smoke is a complex mixture of more than 5000 chemicals and evidence is not clear which components, either alone or in combination, may cause LBW [19]. Nicotine can easily cross the placenta and has been detected in fetal fluids, amniotic fluid, umbilical cord blood, and in placental tissues of pregnant women [20]. Oxidative stress, vasoconstriction, and compromised feto-placental unit function are partly responsible for inadequate nutrient and oxygen supply resulting in slow in utero fetal growth rate and reduced newborn birth weight [21, 22]. Mothers who smoked as little as 10 cigarettes per day throughout pregnancy had a 3-fold increase in odds of giving birth to a newborn with an average weight reduction between 200 g and 377 g, compared to mothers who did not smoke [11–17].

A few prospective population-based studies have been conducted and less is known about the immediate wellbeing of infants delivered at term with LBW. Only a handful of studies considered testing the role of birth weight as a mediator in association with maternal smoking, [23, 24] and none focused on term neonates. To my knowledge, this study is among the first nationally representative studies using mediation to investigate LBW in term neonates as a potential precipitating factor for NICU transfer/admission in relation to maternal smoking intensity status. A novelty in this study was using the previously introduced E-value in place of sensitivity analysis to assess the potential impact of unmeasured confounding necessary to explain away observed associations, so as not to lose statistical significance. It was hypothesized that LBW in term neonates would be most associated with continued high intensity smoking during pregnancy, and that LBW could be a potential mediator linking smoking to immediate newborn NICU transfer/admission. With this background in mind, the first aim of the present study was to estimate the current prevalence of LBW and NICU transfer/admission among term singleton births, and the second aim was to explore LBW as a mediator in the association between maternal smoking intensity exposure status and immediate NICU placement.

## Methods

### Data source and study design

This cross-sectional study was based on the United States Natality File of live births registered in 2016, the first national data of the fully revised 2003 standard birth certificate, deidentified and made publicly available by the National Center for Health Statistics (NCHS), and the Centers for Disease Control and Prevention (CDC) [25, 26]. The entire dataset (*N* = 3,956,112) was restricted to singleton term births (37–41 completed weeks gestation) (*n* = 3,310,631) and data on births less than 37 and more than 41 completed weeks gestation (*n* = 437,332), multiple births, and missing observations (*n* = 208,149; 5.3%) were excluded from analyses. Restricting the sample to singleton term births avoids confounding by gestational age and allows assessment of exposure risk effects separately from other fetal growth pathologies that may differ in severity [27].

### Quality of data

The overall quality of reproductive, maternal and infant health data in the birth certificate was reported as being high, when compared with hospital medical records [28]. Smoking self-reports during pregnancy imply underreporting and nondisclosure or overreporting of cessation and are prone to bias [29, 30]. Almost 25% of pregnant women do not disclose smoking during pregnancy related in part to social stigma [29] and about 42% of women who self-reported abstinence in clinical trials failed biochemical verification [31]. In a study, biochemical assessment of reported smoking was found to have the highest sensitivity and specificity in the 3 months before pregnancy, relative to any trimester of pregnancy [32].

### Definition of variables

#### Exposure variable

Smoking self-reports included trimester of pregnancy and the number of cigarettes (or packs) smoked per day [26]. It was assumed that women who reported smoking were smokers, because nonsmokers would be less likely to report active smoking during pregnancy [33]. The timing of smoking was categorized as early (first and second trimester) and late pregnancy (third trimester), and intensity was categorized as low (< 10 cigarettes smoked per day) and high (≥10 cigarettes smoked per day). As described in a previous study, [18] seven mutually exclusive smoking intensity status categories that capture natural variability in smoking patterns were used to characterize exposure during term pregnancy: (1) *Quitter-Low*: low intensity smoking in early pregnancy only (the first and second trimester only, none in the third trimester); (2) *Quitter-High*: high intensity smoking in early pregnancy only (the first and second trimester only, none in the third trimester); (3) *Maintainer-Low*: low intensity smoking in early pregnancy (first and second trimester) and low intensity smoking in late pregnancy (third trimester); (4) *Maintainer-High*: high intensity smoking in early pregnancy (first and second trimester) and high intensity smoking in late pregnancy (third trimester); (5) *Reducer:* high intensity smoking in early pregnancy (first and second trimester) and low intensity smoking in late pregnancy (third trimester); (7) *Nonsmoker:* no cigarette use in any trimester.

#### Mediator (intermediate variable)

LBW (< 2500 g) was selected as a mediator, because it is considered a marker of poor in utero fetal growth and an indicator of adverse neonatal outcome [34]. A singleton term birth is giving birth to one live infant between 37 0/7 and 41 6/7 weeks gestation [10]. Gestational age was based on the clinical obstetric estimate (OE) that combines information on the date of the last menstrual period and ultrasound measurement for more validity [35].

#### Outcome variable

NICU transport/transfer and NICU admission are two new variables in the revised US standard birth certificate, [26] which in this study were combined into one hybrid variable of NICU transfer/admission.

#### Covariates

Numerous covariates were selected based on prior knowledge and availability in the dataset. Race categories included non-Hispanic White and non-Hispanic Black, ethnicity was Hispanic, and the other race/ethnicity group included non-Hispanic Asian and non-Hispanic American Indian/Alaska Native, based on the 1997 US Office of Management and Budget (OMB) classification standards [36]. Maternal age was categorized as less than 20, 20–24, 25–29, 30–34, or 35 years old and older, and educational attainment categories included less than high school, high school graduate/GED (General Educational Development certificate), some college or associate degree, and a bachelor’s degree or higher. Marital status was married or unmarried, and parity was categorized as multipara if previously delivered at least one child, or nullipara if never delivered a child [37]. Source of payment was private insurance, Medicaid (i.e. a state and federal program that provides health coverage for very low-income individuals), [38] or other forms of payment, such as other government insurance (i.e. Indian Health Services, Champus/Tricare) or self-pay. Prenatal care was based on the trimester in which care began and included early prenatal care if initiated in the first trimester or late/no prenatal care if initiated in the second/third trimester [39].

**Fig. 1:**
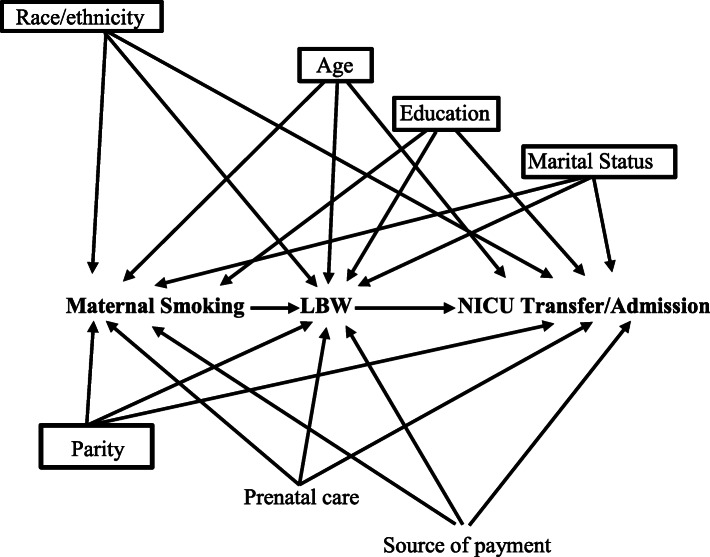
Directed acyclic graph of the hypothesized relationship between maternal smoking intensity status and NICU transfer/admission. Boxed variables are used for adjustment in the model to control for confounding; LBW: low birthweight; NICU: neonatal intensive care unit

Statistical analyses of the data were carried out using SAS version 9.4 (SAS Institute Inc., Cary, NC). The level of statistical significance was set at *p* = 0.05. Initial descriptive analysis included a distribution of maternal smoking intensity exposure status in early (first and second trimester) and in late (third trimester) pregnancy according to demographic characteristics (supplementary information Table 1), and the prevalence of LBW and NICU transfer/admission was estimated across maternal demographic characteristics and smoking intensity status categories (Table 2). Next, as an alternative to the traditional mediation method by Baron and Kenny, [40] a newer mediation technique was used in this study, based on the counterfactual approach [41, 42]. An extended overview of mediation analysis is provided in the Supplementary information. According to the study conceptualization, a small number of selected covariates were needed for adjustment to control for exposure-outcome, exposure-mediator, and mediator-outcome confounding. Multivariable logistic regression analyses, adjusted for maternal race/ethnicity, age, education, marital status, and parity, estimated the odds ratios and corresponding 95% CIs of the total effect, controlled direct effect, the natural direct and indirect effects, and the percentage mediated attributed to LBW, i.e. when 100% of the total effect is mediated (no direct effect) and 0% when there is no mediation (all direct effect). The natural indirect effect was the average difference in NICU transfer/admission of all women who were nonsmokers and delivered an infant with LBW versus all women who were nonsmokers and delivered an infant of normal birth weight. The natural direct effect implied the average difference in the NICU admission if all women smoked and delivered an infant of normal birth weight versus all women who were nonsmokers and delivered an infant of normal birth weight. The outcome in the current study was not common (i.e. the prevalence was < 10%), therefore the odds ratios (ORs) could approximate the relative risk (RRs) ratios [43]. A directed acyclic graph (DAG) (Fig. 1) was constructed based on prior knowledge [11–16, 34] assuming a direct relationship of confounders with the exposure, mediator and outcome variables. Additionally, recently introduced by VanderWeele and Ding, [44, 45] the E-value approach without making assumptions was used in this study in place of sensitivity analysis to assess the potential impact of unmeasured confounding (see supplementary information). The E-value calculations were performed on the estimates and the lower-bounds of the 95% CIs of the estimates [44, 46]. The study sample included the entire population, so weights were not used. This was a secondary analysis of deidentified and publicly available data and the study received Institutional Review Board (IRB) exemption from the University of Maryland.

**Table 1:** Prevalence (% and 95% CI) of low birthweight and neonatal intensive care unit (NICU) transfer/admission across maternal characteristics, singleton term births, United States, 2016

Maternal Characteristics	Low Birthweight *N* = 80,431 (2.4%)	NICU transfer/admission *N* = 148,172 (4.5%)
**Race/ethnicity**		
Non-Hispanic White	1.9 (1.9, 2.0)	4.3 (4.2, 4.3)
Non-Hispanic Black	4.5 (4.5, 4.6)	5.3 (5.3, 5.4)
Hispanic	2.2 (2.1, 2.2)	4.4 (4.3, 4.4)
Other race/ethnicity ^a^	2.9 (2.8, 3.0)	4.7 (4.6, 4.8)
**Maternal Age (years)**		
< 20	3.7 (3.6, 3.8)	5.0 (4.9, 5.0)
20–24	2.9 (2.9, 3.0)	4.5 (4.5, 4.6)
25–29	2.3 (2.2, 2.3)	4.4 (4.3, 4.4)
30–34	2.0 (1.9, 2.0)	4.3 (4.2, 4.3)
35+	2.3 (2.3, 2.4)	4.8 (4.8, 4.9)
**Education**		
Less than high school	3.3 (3.3, 3.4)	4.7 (4.7, 4.8)
High School/GED ^b^	3.0 (2.9, 3.0)	4.7 (4.6, 4.7)
Some college/Assoc.	2.4 (2.3, 2.4)	4.6 (4.6, 4.7)
Bachelor’s or higher	1.7 (1.7, 1.8)	4.1 (4.0, 4.1)
**Marital Status**		
Married	1.9 (1.9, 1.9)	4.0 (4.0, 4.1)
Unmarried	3.3 (3.3, 3.3)	5.2 (5.1, 5.2)
**Parity**		
Nullipara	3.0 (3.0, 3.10)	5.9 (5.8, 5.9)
Multipara	2.0 (2.0, 2.1)	3.6 (3.6, 3.6)
**Form of payment**		
Private Insurance	1.9 (1.8, 1.9)	4.2 (4.1, 4.2)
Medicaid	3.2 (3.1, 3.2)	4.9 (4.9, 5.0)
Other forms of payment ^c^	2.2 (2.2, 2.3)	4.0 (3.9, 4.1)
**Prenatal Care**^**d**^		
Early	2.2 (2.2, 2.3)	4.3 (4.2, 4.3)
Late/no care	3.1 (3.0, 3.1)	5.2 (5.2, 5.3)

*CI* Confidence interval; ^a^ Other race/ethnicity: non-Hispanic Asian, non-Hispanic American Indian Alaska Native, non-Hispanic Native Hawaiian or other Pacific Islander, mixed race; ^b^ GED: general educational development; ^c^ Other forms of payment: self-pay, Indian Health Service, Champus/Tricare, or other government insurance; ^d^ Early prenatal care if initiated in the first trimester or late/no prenatal care if initiated in the second/third trimester

**Table 2:** Prevalence of low birthweight and neonatal intensive care unit (NICU) transfer/admission according to maternal smoking intensity exposure status

Maternal smoking intensity status	Low birthweight N = 80,431 (2.4%) % (95% CI)	NICU transfer/admission N = 148,172 (4.5%) % (95% CI)
Quitter-Low	3.6 (3.4, 3.8)	5.3 (5.0, 5.5)
Quitter-High	3.7 (3.4, 4.0)	5.8 (5.5, 6.2)
Maintainer-Low	5.4 (5.2, 5.6)	6.1 (5.9, 6.3)
Maintainer-High	6.7 (6.5, 6.9)	7.0 (6.8, 7.2)
Reducer	5.8 (5.6, 6.1)	6.3 (6.0, 6.6)
Increaser	4.9 (3.8, 5.9)	6.4 (5.1, 7.6)

*CI* confidence interval; Low intensity smoking: < 10 cigs/day; High intensity smoking: ≥10 cigs/day

## Results

### Overall prevalence of LBW and NICU transfer/admission

As shown in Table 1, among singleton term births (*N* = 3,310,631) in 2016, the LBW rate was 2.4% and the rate of NICU transfer/admission was 4.5%, and both were most prevalent among non-Hispanic Black women (4.5 and 5.3%, respectively), less than 20 years old (3.7 and 5.0%, respectively), and with less than a high school education (3.0 and 4.7%, respectively). Although lower, the rates were also substantial among unmarried, nulliparas, women on Medicaid and with late/no prenatal care.

### Distribution by smoking intensity status

Nearly 6.8% of women who gave birth to a singleton infant at term smoked in early (first and second trimester) and in late (third trimester) pregnancy, and most (36.4%) smoked at high intensity (≥ 10 cigarettes /day) (Supplementary Table 1). The highest percentage distribution (over 40%) of high intensity smoking in early and in late pregnancy was among non-Hispanic White women, 35+ years old, and women with less than high school education. While somewhat lower, high intensity smoking was noteworthy among women who were married, multiparas, with late/no prenatal care, and on Medicaid.

### Prevalence of LBW and NICU transfer/admission across smoking intensity exposure status

The prevalence of LBW varied by smoking intensity exposure status (Table 2) ranging from 3.6% among low intensity quitters in early pregnancy only (*Quitters-Low*) to 6.7% among continued high intensity smokers in early and late pregnancy (*Maintainers-High*). Likewise, the prevalence of NICU transfer/admission ranged from 5.3% among low intensity quitters in early pregnancy to 7.0% among high intensity continued smokers (maintainers).

### Mediation analysis

The estimates of the total effect, controlled effect, the natural direct and natural indirect effects in association with each smoking intensity status category on NICU admission were statistically significant and greater than 1 (Table 3). A direct effect greater than 1 indicated that the risk of NICU transfer/admission was higher not including the effect of LBW, and an indirect effect greater than 1 indicated that the risk of smoking was higher and LBW increased the risk of NICU admission. The estimated controlled direct effect aOR was also greater than 1 and it was similar to the natural direct effect. These findings suggested that LBW mediated the association of each maternal smoking intensity category with NICU transfer/admission (Table 3). The effect of high intensity smoking exposure status (*Maintainers-High)* had the highest percentage mediated attributed to LBW (17.58%), followed by *Reducers* (15.6%), *Quitters-High* and *Quitters-Low* (13.89 and 13.35%, respectively), and *Increasers* (12.91%).

**Table 3:** Adjusted odds ratios for neonatal intensive care unit admission associated with total effect, controlled direct, natural direct and natural indirect effects, and percentage mediated through low birthweight

Smoking Intensity Status	Total Effect		Controlled Direct Effect		Natural Direct Effect		Natural Indirect Effect		Percentage Mediated	
	aOR	95% CI	aOR	95% CI	aOR	95% CI	aOR	95% CI	%	95% CI
Quitter-Low	1.14	1.08, 1.19	1.13	1.07, 1.19	1.12	1.06, 1.18	1.02	1.01, 1.02	13.35	6.66, 20.04
Quitter-High	1.28	1.20, 1.37	1.24	1.15, 1.33	1.24	1.16, 1.33	1.03	1.02, 1.04	13.89	8.33, 19.45
Maintainer-Low	1.38	1.34, 1.43	1.35	1.30, 1.40	1.33	1.28, 1.37	1.04	1.04, 1.05	15.14	12.52, 17.77
Maintainer-High	1.68	1.63, 1.73	1.58	1.53, 1.63	1.56	1.51, 1.61	1.08	1.07, 1.09	17.58	15.48, 19.67
Reducer	1.44	1.37, 1.51	1.40	1.33, 1.47	1.37	1.31, 1.44	1.05	1.04, 1.06	15.58	11.81, 19.36
Increaser	1.48	1.18, 1.79	1.44	1.13, 1.76	1.42	1.12, 1.72	1.04	1.00, 1.09	12.91	−0.91, 26.73

*aOR* adjusted odds ratio, *CI* confidence interval; Low intensity smoking: < 10 cigarettes/day; High intensity smoking: ≥10 cigs/day; Adjusted for: maternal race/ethnicity, age, education, marital status, and parity. Total effect = natural direct + natural indirect effect

### E-value assessment

For the high intensity smoking exposure (*Maintainer-High*), the estimated size of the total effect was 1.68 and the E-value was 2.8, i.e. the total effect could be explained away by unmeasured confounding associated with both, smoking exposure and NICU transfer/admission by an odds ratio of 2.8-fold each or larger. Likewise, the estimated size of the natural direct and indirect effects were 1.56 and 1.08, respectively, and their corresponding E-values were of 2.5 and 1.4, respectively, i.e. the effects could be explained away by unmeasured confounding by an odds ratio of 2.5-fold and 1.4-fold, respectively, above and beyond any measured confounding, but weaker confounding could not do so. E-value calculations based on the lower-bounds of the 95% confidence intervals (CIs) of the effect estimates from the largest smoking exposure category (*Maintainers-High)* were 2.6, 2.4, and 1.8 for the total effect, natural direct and the natural indirect effects, respectively, and for the smallest exposure category (*Quitters-Low*) were 1.4, 1.3, and 1.1, respectively. In brief, the observed associations could lose statistical significance by shifting the odds toward the null, if unmeasured confounding was associated with *Maintainers-High* and NICU transfer/admission by a relative effect of 2.6, 2.4, and 1.8, and if associated with *Quitters-Low* and NICU transfer/admission by a relative effect of 1.4, 1.3, and 1.1, respectively, beyond the observed covariates.

## Discussion

Neonates at term receive less attention in research and this study fills a gap on the significance of LBW in precipitating immediate NICU transfer/admission. This is one of the first studies to report data on the prevalence of LBW and NICU transfer/admission in the US in 2016 among singleton term births across a set of smoking intensity exposure status categories in early and in late pregnancy, and to investigate the role of LBW linking smoking to immediate newborn NICU placement. By addressing the prevalence and mediation effects of LBW, this study draws attention to burden of illness among term neonates of mothers who continue smoking throughout pregnancy. An estimated 6.8% of women smoked in early and in late pregnancy, which is in line with other reports [9]. In support of the study hypothesis, persistent high intensity smoking in early and in late pregnancy was found to be associated with the highest rates of LBW and NICU transfer/admissions in term neonates. The etiology of LBW is multifactorial and maternal, placental, and fetal risk factors are acting together in different trimesters of pregnancy to influence fetal growth and newborn birth weight [11–16, 22]. A gestational vulnerability window for smoking exposure argues for early pregnancy, when risks for preterm birth [47] and low birthweight [48] are greatly increased. Almost 13.8% of women who reduced their smoking intensity from early to late pregnancy [18] might have improved their odds for preterm birth and deficit in newborn weight, relative to women who continued smoking at high intensity [49, 50].

Only a few prior studies considered LBW as a mediator and the implicated mechanisms are not completely understood. For instance, Gold et al. [23] examined LBW and preterm birth as mediators in association with stillbirth, and Geraci and Mattei, [24] investigated the risk of sudden infant death syndrome using birth weight as a mediator. The current study was able to show that exposure to high intensity smoking during a term gestation could influence birth weight and newborn health status, which was not apparent in previous research. LBW in term neonates was a mediator in association with every maternal smoking intensity status category and contributed to an increased risk of NICU transfer/ admission. Because nearly one-fifth (17.58%) of the total effect size estimate on NICU admission was attributed to LBW in association with persistent high intensity smoking, it is assumed that the risk can occur through other pathways independent of LBW. While the change may seem small, nevertheless, for heavier maternal smokers, almost 1 in 5 of all NICU admissions could be attributed to LBW in term neonates. Whereas for lighter smokers, quitters and reducers, although the risk of LBW and NICU admission remained lower, it was still meaningful. Considering that there are almost 4 million births each year in the United States and about 90% of them are term births, morbidity in this large cohort of infants may be a substantial load on resource utilization and healthcare costs. Confounding related to variability in magnitude of smoking exposure in each trimester of term pregnancy could bias direct and indirect effect estimates making sensitivity analyses quite complex. In the current study, a simpler E-value technique was used in place of sensitivity analysis to assess the potential impact of unmeasured confounding, conditional on measured covariates [44–46]. The calculations were based on the effect size estimates and on the lower bounds 95% CIs, to account for uncertainty (see supplemental information). Given a large sample size and relatively small E-values, it was assumed that the exposure category with larger estimates (*Maintainer-High*) will be more robust to unmeasured confounding, when compared to a category with smaller estimates (*Quitters-Low*) [44–46]. In fact, E-values were relatively small in both fields implying that little unmeasured confounding could explain away most of the observed associations in this study, in the absence of other forms of bias.

Important implications of the current study include underpinnings of methodological research on adverse effects of smoking behavior in pregnancy affecting wellbeing of term neonates in the United States. Smoking is a primary pathway through which resource utilization and healthcare costs can be significantly influenced [51]. Thus, early identification of mothers who smoke and close monitoring of term pregnancy in preparation for delivery is imperative. Moreover, this study opens an opportunity for future research on the significance of neonatal morbidity across the whole spectrum of term births (i.e. early-term, full-term and late-term).

### Strengths and limitations

The main strength of the current study was availability of data from the fully revised US Standard Birth Certificate [26] that provided new variables and allowed findings to be nationally representative. Second, smoking exposure was well characterized in this study by restricting the sample to singleton term births and using a set of novel smoking intensity status categories to capture the spectrum of natural variability in maternal smoking patterns. Third, very few prior studies applied statistical mediation based on the counterfactual approach to shed light on mechanisms underlying immediate morbidity in infants, independent of gestational age. Finally, a novelty in this population-based study was using the recently introduced E-value as an alternative to sensitivity analysis. There are also some limitations. A retrospective cross-sectional design does not establish temporality. Smoking self-reports that were not biochemically validated may be considered biased due to underreporting and nondisclosure, or overreporting of cessation [29, 30]. Information on month-specific and non-daily smoking (< 1 cigarette/day) in pregnancy is not available on the birth certificate. Effect size estimates may be affected by different forms of reporting bias. Because it is assumed that nondifferential measurement error and misclassification will bias toward the null, the reported E- values may create some underestimates of confounding [46].

## Conclusions

The findings of this study could be used to update practitioners on additional support to term infants of mothers who smoked in pregnancy, to monitor trends, and to inform planning and allocation of healthcare resources. Concerns about LBW and morbidity in neonates at term should be included in smoking cessation interventions targeting all women before, during and after pregnancy.

## Supplementary information

**Additional file 1 Table S1.** Distribution of smoking intensity status in early and late pregnancy according to maternal characteristics, singleton term births (37–41 completed weeks gestation), United States, 2016 **Supplementary information** Overview of mediation analysis and the E-value approach, and additional Table1 are provided in the Supplementary information.

## Data Availability

The dataset analyzed during the current study is publicly available from the National Center for Health Statistics (NCHS) Vital Statistics Natality Birth Data and can be accessed through the National Bureau of Economic Research website: http://www.nber.org/data/vital-statistics-natality-data.html.

## Abbreviations

aOR: Adjusted odds ratio
CDE: Controlled direct effect
CI: Confidence interval
DAG: Directed acyclic graph
LBW: Low birthweight
NCHS: National Center for Health Statistics System
NDE: Natural direct effect
NICU: Neonatal intensive care unit
NIE: Natural indirect effect
TE: Total effect
