# Supplementary material for: Low birthweight in term singletons mediates the association between maternal smoking intensity exposure status and immediate neonatal intensive care unit admission: the E-value assessment
Source: BMC Pregnancy Childbirth. 2020 Jun 3;20:341. doi: 10.1186/s12884-020-02981-1 (PMC7268400; doi:10.1186/s12884-020-02981-1)
Supplement: Supplementary file 1 — Additional file 1 Table S1. Distribution of smoking intensity status in early and late pregnancy according to maternal characteristics, singleton term births (37–41 completed weeks gestation), United States, 2016 Supplementary information Overview of mediation analysis and the E-value approach, and additional Table1 are provided in the Supplementary information. [file 12884_2020_2981_MOESM1_ESM.docx]

**TITLE:** Low birthweight in term singletons mediates the association between maternal smoking intensity exposure status and immediate neonatal intensive care unit admission: The E-value assessment

**AUTHOR:** Anthony J. KONDRACKI, MD, MPH, PhD, CPH (email: akondrac@umd.edu)

**SUPPLEMENTARY INFORMATION**

**Mediation analysis**

Low birthweight (LBW) in term neonates was a potential mediator in the association between maternal smoking intensity status and NICU transfer/admission. As an alternative to the traditional method for mediation by Baron and Kenny, [40] this study followed a newer technique based on the counterfactual approach. The counterfactual approach defines mediation effects in terms of counterfactuals to account for potential confounders and allowing a decomposition of the total effect (TE) (i.e. the overall effect of exposure on the outcome) into the natural direct effect (NDE) and the natural indirect effect (NIE), while operating through the mediator. [41] [42] The advantage of the natural effects is that estimates are valid even if the effect of exposure on the outcome differs by the level of the mediator (e.g. LBW vs. normal birth weight).[41] [42] The controlled direct effect (CDE) is similar to the NDE if smokers or nonsmokers delivered an infant of normal or LBW. This method is able to capture potential interactions between the exposure, mediator, and outcome that describe associations between maternal smoking intensity exposure status, LBW, and NICU transfer/admission in newborn at term. [42] The necessary assumptions for a causal mediation analysis included: no unmeasured confounding between exposure and outcome, exposure and mediator, and mediator and outcome, and no confounding between mediator and outcome produced by exposure. [42] All assumptions were satisfactory for our study aims to allow for data interpretation.

**The E-value approach**

The E-value is a measure (on the risk ratio scale) of the minimum strength of the association that an unmeasured confounder would need to have with the exposure and outcome to explain away a specific association given measured confounders, which were adjusted for in analyses. [44] Because it was assumed that high intensity smoking exposure status in early and in late pregnancy (*Maintainer-High*) will yield the largest effect size estimate, this exposure category was selected for the E-value calculation. To assess uncertainty to bias further, the E-value calculation was also performed on the lower-bounds of the 95% CIs of effect estimates of the two most extreme smoking intensity status categories i.e. the largest (*Maintainers-High*) and the smallest (*Quitters -Low*), for comparison.[44] [46]

**Supplementary Table 1.**

| **Table 1.** Distribution of smoking intensity status in early and late pregnancy according to maternal characteristics, singleton term births (37-41 completed weeks gestation), United States, 2016 | | | | | | | | |
| --- | --- | --- | --- | --- | --- | --- | --- | --- |
|  |  | **QUITTER-LOW** | **QUITTER-HIGH** | **MAINTAINER-LOW** | **MAINTAINER-HIGH** | **REDUCER** | **INCREASER** |  |
| **Maternal Characteristics** | **Smoking in Early and Late pregnancy** | **Low intensity smoking in Early pregnancy only** | **High intensity smoking in Early pregnancy only** | **Low intensity smoking in Early and Low intensity smoking in Late pregnancy** | **High intensity smoking in Early and High intensity smoking in Late pregnancy** | **High intensity smoking in Early and Low intensity smoking in Late pregnancy** | **Low intensity smoking in Early and High intensity smoking in Late pregnancy** |  |
| **Total % (95% CI)** | 6.8 (6.8, 6.9) | 13.0 (12.9, 13.2) | 7.2 (7.0, 7.3) | 28.7 (28.6, 29.0) | 36.4 (36.2, 36.6) | 14.0 (13.8,14.1) | 0.7 (0.7, 0.7) |  |
| **Race/ethnicity** |  |  |  |  |  |  |  |  |
| Non-Hispanic White | 77.4 (77.2, 77.6) | 11.1 (10.9, 11.2) | 7.3 (7.1, 7.4) | 25.3 (25.2, 25.7) | 40.7 (40.4, 40.9) | 14.9 (14.7, 15.1) | 0.7 (0.7, 0.7) |  |
| Non-Hispanic Black | 10.9 (10.8, 11.0) | 18.8 (18.3, 19.2) | 5.8 (5.5, 6.1) | 45.4 (44.8, 46.1) | 20.3 (19.8, 20.8) | 9.1 (8.8, 9.5) | 0.6 (0.5, 0.7) |  |
| Hispanic | 5.7 (5.6, 5.8) | 24.1 (23.3, 24.8) | 8.7 (8.2, 9.2) | 36.1 (35.3, 36.9) | 19.6 (18.9, 20.3) | 10.9 (10.4, 11.5) | 0.6 (0.5, 0.7) |  |
| Other race/ethnicity ^a^ | 6.0 (5.9, 6.0) | 17.7 (17.1, 18.4) | 7.4 (7.0, 7.8) | 34.5 (33.7, 35.3) | 26.1 (25.4, 26.8) | 13.6 (13.0, 14.2) | 0.7 (0.6, 0.9) |  |
| **Age (years)** |  |  |  |  |  |  |  |  |
| <20 | 5.3 (5.3, 5.3) | 18.6 (18.0, 19.3) | 8.8 (8.3, 9.2) | 31.1 (30.4, 31.9) | 26.2 (25.5, 26.9) | 14.5 (13.9, 15.0) | 0.8 (0.7, 1.0) |  |
| 20-24 | 19.8 (19.7, 19.8) | 14.6 (14.4, 14.9) | 7.6 (7.4, 7.8) | 29.7 (29.3, 29.9) | 32.6 (32.3, 33.0) | 14.9 (14.6, 15.2) | 0.6 (0.6, 0.7) |  |
| 25-29 | 29.2 (29.2, 29.3) | 12.2 (11.9, 12.4) | 6.7 (6.5, 6.8) | 28.3 (28.0, 28.7) | 38.2 (37.9, 38.6) | 14.0 (13.8, 14.3) | 0.6 (0.6, 0.7) |  |
| 30-34 | 28.8 (28.8, 28.9) | 11.2 (10.9, 11.5) | 6.9 (6.7, 7.1) | 27.9 (27.5, 28.4) | 40.3 (39.8, 40.7) | 13.0 (12.7, 13.3) | 0.7 (0.6, 0.7) |  |
| 35+ | 16.9 (16.8, 16.9) | 10.8 (10.4, 11.2) | 7.1 (6.8, 7.5) | 27.7 (27.1, 28.3) | 41.4 (40.7, 42.0) | 12.2 (11.8, 12.6) | 0.8 (0.7, 0.9) |  |
| **Education** |  |  |  |  |  |  |  |  |
| Less than high school | 24.2 (24.0, 24.3) | 9.6 (9.3, 9.8) | 5.3 (5.1, 5.5) | 29.9 (29.5, 30.3) | 40.8 (40.4, 41.2) | 13.7 (13.4, 14.0) | 0.7 (0.7, 0.8) |  |
| High School/GED ^b^ | 41.7 (41.6, 42.0) | 12.2 (12.0 ,12.4) | 6.9 (6.7, 7.0) | 28.6 (28.4, 28.9) | 37.5 (37.2, 37.8) | 14.1 (13.9, 14.4) | 0.7 (0.6, 0.7) |  |
| Some college/Assoc. | 30.6 (30.4, 30.8) | 15.7 (15.4, 16.0) | 8.6 (8.4, 8.8) | 28.0 (27.7, 28.3) | 32.6 (32.3, 33.0) | 14.4 (14.1, 14.6) | 0.7 (0.6, 0.7) |  |
| Bachelor’s or higher | 3.5 (3.4, 3.6) | 23.8 (22.9, 24.7) | 11.1 (10.4, 11.8) | 29.6 (28.6, 30.6) | 24.9 (23.9, 25.8) | 9.9 (9.2, 10.6) | 0.7 (0.5, 0.9) |  |
| **Marital Status** |  |  |  |  |  |  |  |  |
| Married | 27.9 (27.7, 28.1) | 12.9 (12.6, 13.1) | 7.3 (7.1, 7.5) | 26.9 (26.6, 27.2) | 39.2 (38.8, 39.6) | 12.9 (12.7, 13.2) | 0.8 (0.7, 0.8) |  |
| Unmarried | 72.1 (71.9, 72.3) | 13.1 (12.9. 13.3) | 7.1 (7.0, 7.3) | 29.5 (29.3, 29.7) | 35.3 (35.0, 35.5) | 14.4 (14.2, 14.5) | 0.6 (0.6, 0.7) |  |
| **Parity** |  |  |  |  |  |  |  |  |
| Nullipara | 30.3 (30.1, 30.5) | 18.8 (18.5, 19.1) | 10.1 (9.8, 10.3) | 28.0 (27.7, 28.3) | 27.6 (27.3, 28.0) | 14.8 (14.5, 15.0) | 0.7 (0.6, 0.7) |  |
| Multipara | 69.7 (69.5, 69.9) | 10.5 (10.4, 10.7) | 5.9 (5.8, 6.0) | 29.1 (28.9, 29.3) | 40.2 (39.9, 40.4) | 13.6 (13.4, 13.8) | 0.7 (0.6, 0.7) |  |
| **Prenatal Care ^c^** |  |  |  |  |  |  |  |  |
| Early | 65.7 (65.5, 65.9) | 13.9 (13.7, 14.1) | 7.4 (7.3, 7.6) | 28.9 (28.6, 29.1) | 35.7 (35.4, 35.9) | 13.4 (13.3, 13.6) | 0.7 (0.7, 0.8) |  |
| Late/no care | 34.3 (34.1, 34.5) | 11.4 (11.2, 11.6) | 6.7 (6.5, 6.9) | 28.6 (28.3, 28.9) | 37.8 (37.4, 38.1) | 14.9 (14.7, 15.2) | 0.6 (0.5, 0.6) |  |
| **Source of Payment** |  |  |  |  |  |  |  |  |
| Private Insurance | 21.4 (19.4, 19.8) | 18.1 (17.7, 18.4) | 10.0 (9.7, 10.2) | 28.3 (27.9, 28.7) | 30.3 (29.9, 30.7) | 12.6 (12.3, 12.9) | 0.7 (0.6, 0.7) |  |
| Medicaid | 73.9 (73.7, 74.0) | 11.5 (11.4, 11.7) | 6.3 (6.2, 6.4) | 29.0 (28.7, 29.2) | 38.2 (37.9, 38.4) | 14.3 (14.2, 14.5) | 0.7 (0.6, 0.7) |  |
| Other forms of payment ^d^ | 4.7 (4.7, 4.9) | 14.1 (13.4, 14.7) | 8.2 (7.7, 8.7) | 28.0 (27.1, 28.8) | 35.7 (34.8, 36.6) | 13.3 (12.7, 14.0) | 0.7 (0.5, 0.9) |  |

Early pregnancy: first and second trimester; Late pregnancy: 3rd trimester; Low intensity smoking: <10 cigs/day; High intensity smoking ≥10 cigs/day;  ^a^ Other race/ethnicity: non-Hispanic Asian, non-Hispanic American Indian Alaska Native, non-Hispanic Native Hawaiian, Pacific Islander, or mixed race; ^b^ GED: general educational development; ^c^ Early prenatal care if initiated in the first trimester or late/no prenatal care if initiated in the second trimester or later; ^d^ Other forms of payment: self-pay, Indian Health Service, Champus/Tricare, or other government insurance.
